# Principles of Forensic Medicine

**Published:** 1845-03

**Authors:** 


*Principles of Forensic Medicine.* By WILLIAM A. GUY, M. B., Cantab., Fellow of the Royal College of Physicians; Professor of Forensic Medicine, King's College, London, &c., &c. First American edition, with notes and additions. By CHARLES LEE, M. D., Professor of Materia Medica and General Pathology in Geneva College, &c., &c. pp. 711. New York, HARPER & BROTHERS.

THIS is a valuable and extensive summary of medical jurisprudence, carefully made by a gentleman well qualified by position and ability to do justice to the subject.

The author has omitted all details of purely literary interest, and avoided all discussion of obsolete and foreign laws; his aim being to make the book as practical as possible.

The American editor has added very greatly to the value of the English edition, by the extensive and valuable additions which he has made to it; and by adapting it to the "laws and institutions of this country."

As physicians are constantly liable to be called upon to testify in courts of justice, and as much importance is deservedly attached to their evidence, it is of great consequence to every one of them to have a manual by which they may be guided in preparing to give the best possible information to the court and jury. To all we can recommend the work of Dr. Guy.—*Balt. Editor.*

